# Independent risk factors for disease recurrence after surgery in patients with hepatitis B virus-related hepatocellular carcinoma ≤3 cm in diameter

**DOI:** 10.1093/gastro/goz009

**Published:** 2019-03-30

**Authors:** Ling-Ling He, Xiao-Li Liu, Shuan Zhang, Meng-Ge Li, Xian-Bo Wang, Yu-Yong Jiang, Zhi-Yun Yang

**Affiliations:** Center of Integrative Medicine, Beijing Ditan Hospital, Capital Medical University, Beijing, P.R. China

**Keywords:** Hepatitis B virus, hepatocellular carcinoma, prognostic factors, recurrence

## Abstract

**Background:**

Post-operative recurrence rates are high for hepatitis B virus (HBV)-related hepatocellular carcinoma (HCC). This study aimed to explore the factors associated with post-operative 1-year recurrence rate in patients with HBV-related HCC who had a single small primary tumor (≤3 cm in diameter).

**Methods:**

This was a retrospective study of 203 (training cohort) and 64 (validation cohort) patients newly diagnosed with HBV-related HCC who had a single small primary tumor. The first year of post-operative follow-up was examined. Factors potentially associated with HCC recurrence were identified using Cox regression analyses. A model was constructed based on the factors identified and the prognostic value of the model was evaluated using receiver operating characteristic (ROC) curve analysis and calculation of the area under the ROC curve (AUC).

**Results:**

A history of alcoholism and serum levels of α-fetoprotein, total protein and γ-glutamyl transpeptidase (GGT) were independently associated with 1-year recurrence rate after surgery. A predictive model based on these four factors had an AUC of 0.711 (95% confidence interval, 0.643–0.772) in the training cohort and 0.727 (95% confidence interval, 0.601–0.831) in the validation cohort. The 1-year recurrence rate was significantly lower in the low-risk group than in the high-risk group in both the training cohort (17.0% vs. 49.5%, *P *<* *0.001) and the validation cohort (43.2% vs. 74.1%, *P *=* *0.031).

**Conclusion:**

A history of alcoholism and serum levels of α-fetoprotein, total protein and γ-glutamyl transpeptidase were independently associated with post-operative 1-year recurrence rate in patients with HBV-related HCC who had a single small primary tumor (≤3 cm in diameter).

## Introduction

In 2012, approximately 78.25 million people worldwide were diagnosed with hepatocellular carcinoma (HCC) and 74.55 million people died from this cancer [1]. Chinese HCC patients account for more than 50% of all newly diagnosed cases of HCC and all deaths due to HCC [1]. Chronic hepatitis B virus (HBV) infection is a major risk factor for HCC [2]. About 350 million people worldwide suffer from chronic HBV infection and HBV infection is the main cause of HCC in China [3, 4].

The detection rate of small primary HCC has increased in recent years due to improvements in imaging techniques [5–7]. However, disease recurrence commonly occurs after the treatment of small HCCs, with 1-year recurrence rates reported to be 30%–40% [8–10]. Many factors affect the risk of post-operative HCC recurrence, including tumor size, tumor encapsulation, microvascular invasion, liver cirrhosis, serum α-fetoprotein (AFP) level >400 μg/L and use of antiviral drugs [11–14].

Various scoring systems are available for predicting the risk of primary HCC occurrence in Chinese and other populations [15–19]. Nevertheless, HBV-related HCC has a natural history that differs from that of non-HBV-related HCC [3, 4]. Furthermore, prognosis is influenced by whether there is early or late recurrence of HCC after surgical resection of the primary tumor [20, 21]. Currently, there is no scoring system to predict patient survival or the recurrence of HBV-related HCC after treatment in patients with a small primary tumor. Such a scoring system could facilitate close surveillance of patients at high risk of recurrence.

There is controversy regarding the definition of early versus late recurrence of HCC. Park *et al*. [22] observed that the survival rate of patients who had HCC recurrence within 6 months of liver resection was significantly lower than that of patients who experienced HCC recurrence after 6 months. Therefore, they suggested a time point of 6 months after treatment as a threshold for early/late recurrence. On the other hand, Cheng *et al*. [23] suggested that recurrence within 2 years after surgery should be considered early recurrence. Guidelines generally recognize the 2-year threshold for early/late recurrence, based on the concept that early recurrence (<2 years) is caused by intrahepatic metastases, whereas late recurrence (>2 years) is caused by multicentric metastases [24]. Nevertheless, Imamura *et al*. [25] suggested that the first post-operative year was the period in which the risk of HCC recurrence was the highest. Based on the characteristics of the patients (geographical location [Asia] and tumor size), the 1-year threshold was selected for use in the present study.

This study aimed to explore the factors associated with post-operative 1-year recurrence rate in patients with HBV-associated HCC who had a single small (≤3 cm in diameter) primary tumor and to develop a scoring system that could predict the risk of recurrence. It was anticipated that the results would provide a basis for clinicians to determine the post-operative recurrence risk of HCC.

## Methods

### Study design

This was a retrospective study of patients with HBV-related HCC who had a single small (≤3 cm) primary tumor. All patients were grouped into two cohorts. The training and validation cohorts included patients newly diagnosed with HBV-related HCC and a single small primary tumor at Beijing Ditan Hospital, Capital Medical University (Beijing, China) between January 2012 and December 2014 and between January 2015 and June 2015, respectively.

The study was approved by the ethics committee of Beijing Ditan Hospital, Capital Medical University. The need for individual consent was waived by the committee because of the retrospective nature of the study.

### Patient inclusion

The inclusion criteria were as follows: (i) HBV-related HCC; (ii) a single isolated small (≤3 cm in diameter) primary tumor; (iii) 18–75 years of age; and (iv) data were available for at least 1 year of follow-up after surgery. The exclusion criteria included the following items: (i) evidence of hepatitis C virus (HCV) or human immunodeficiency virus (HIV) infection; (ii) severe disease or dysfunction of the heart, lungs, brain, kidneys or other vital organs; (iii) severe mental illness; (iv) pregnancy/lactation; or (v) incomplete clinical data.

For patients who underwent surgery or biopsy, the diagnosis of HCC was based on histopathological examination. For patients who did not undergo biopsy or surgery, the diagnosis of HCC was made clinically according to the clinical symptoms and either at least two imaging modalities indicating HCC (hepatic arteriography, magnetic resonance imaging [MRI], computed tomography [CT] and liver ultrasound) or one imaging modality indicating HCC combined with a serum AFP level ≥400 ng/mL [2].

The patients were treated according to international guidelines [24]. Based on the Chinese guidelines, transarterial chemoembolization (TACE) was used as a locoregional treatment because of the minimal trauma associated with this technique [26]. Blood tests, measurements of HBV-DNA and serum AFP, and liver ultrasonography were performed every 3–6 months after surgery [24, 26].

### Outcomes

The analysis was limited to the first year of follow-up after surgery. The outcome was recurrence of HCC during the first year after surgery. Measurements of serum AFP level and liver ultrasonography were carried out every 3–6 months. CT or MRI was performed if necessary. The criteria used to diagnose HCC recurrence were: (i) new lesions observed in the original tumor bed and its surroundings or other parts of the liver; and (ii) the new lesions met the imaging criteria for primary HCC [2].

### Collection of clinical data

The following baseline parameters and outcome factors were extracted from the medical records: sex, age, surgical approach, smoking history, history of alcoholism, family history, tumor size, cirrhosis, white blood cell count (WBC), CD8^+^ lymphocytes, hemoglobin (HGB) level, neutrophil–lymphocyte ratio (NLR), serum levels of alanine transaminase (ALT), aspartate aminotransferase (AST), γ-glutamyl transpeptidase (GGT), total bilirubin (TBIL), total protein (TP), albumin (ALB), creatinine (Cr) and AFP, prothrombin time (PT), HBV-DNA, Child-Turcotte-Pugh (CTP) score and Model for End-Stage Liver Disease (MELD) score [27]. The reference range for AFP (ARCHITECT AFP Assay, Abbott Laboratories, IL, USA) was 0.89–8.78 ng/mL. Therefore, a serum AFP level >8.78 ng/mL was considered a positive test result.

### Statistical analysis

Statistical analysis was performed using SPSS 19.0 (IBM Corp., Armonk, NY, USA). Data were tested for normality by the Kolmogorov-Smirnov method. Normally distributed continuous data are expressed as mean ± standard deviation and were analyzed using Student’s *t*-test. Non-normally distributed continuous data are presented as median (range) and were analyzed using the Mann–Whitney *U* test. Categorical data are expressed as frequency and were analyzed using the chi-squared test.

First, factors associated with the 1-year recurrence rate were analyzed by univariable Cox regression analyses. Then, factors with *P*-values <0.05 were included in a multivariable Cox regression analysis (using the forward and maximal-likelihood ratio methods) to establish a Cox proportional hazards regression model. Recurrence rate was analyzed by the Kaplan–Meier method and the log-rank test. The cut-off values for each factor and for the whole model were determined by receiver operating characteristic (ROC) curve analysis with calculation of the Youden index and the diagnostic value of the established model was evaluated using the area under the ROC curve (AUC). Cut-offs for continuous variables were based on the Youden index. A two-sided *P*-value <0.05 was considered statistically significant.

## Results

### Baseline characteristics

Patients with evidence of HCV or HIV infection (*n *=* *42), with severe disease or dysfunction of the heart, lungs, brain, kidneys or other vital organs (*n *=* *4), with severe mental illness (*n *=* *2), who were pregnant or breastfeeding (*n *=* *1) or with incomplete clinical data (*n *=* *35) were excluded from the study.

The training cohort included 203 patients, 149 males (73.4%) and 54 females (26.6%), with a mean age of 54.7 ± 9.0 years. The validation cohort included 64 patients, 47 males (73.4%) and 17 females (26.6%), with a mean age of 57.1 ± 9.2 years.

The number of patients with HCC recurrence during the first year after treatment was 66 patients (32.5%) in the training cohort and 36 patients (56.3%) in the validation cohort (Table 1).

**Table 1.** Baseline characteristics of patients with a primary single small HBV-associated HCC according to the recurrence status at 1 year

	Training cohort			Validation cohort		
Characteristic	Recurrence (*n* = 66)	No recurrence (*n* = 137)	*P*	Recurrence (*n* = 36)	No recurrence (*n* = 28)	*P*
Age^a^ (years)	54.9 ± 9.1	54.4 ± 9.0	0.723	58.2 ± 9.1	55.6 ± 9.3	0.262
Sex (male)	54 (81.8%)	95 (69.3%)	0.060	30 (83.3%)	17 (60.7%)	0.042
Drinking history (>20 g/day)	24 (36.4%)	30 (21.9%)	0.029	18 (50.0%)	6 (21.4%)	0.019
Smoking history	23 (34.8%)	38 (28.1%)	0.332	13 (36.1%)	7 (25.0%)	0.341
Family history	24 (36.4%)	37 (27.0%)	0.173	14 (38.9%)	10 (35.7%)	0.795
CTP grade			0.025			0.182
A	37 (56.1%)	86 (62.8%)		18 (50.0%)	20 (71.4%)	
B	18 (27.3%)	44 (32.1%)		11 (20.6%)	6 (21.4%)	
C	11 (16.7%)	7 (5.1%)		7 (19.4%)	2 (7.1%)	
Liver cirrhosis	62 (93.9%)	126 (91.9%)	0.300	32 (88.9%)	25 (89.3%)	0.960
Portal-vein thrombosis and/or vascular invasion	2 (3.0%)	3 (2.2%)	0.717	1 (2.8%)	1 (3.6%)	0.856
Portal hypertension	15 (22.7%)	39 (28.5%)	0.386	12 (33.3%)	5 (17.9%)	0.164
Tumor size			0.577			0.355
<2 cm	36 (54.5%)	69 (50.4%)		19 (52.8%)	18 (64.3%)	
2–3 cm	30 (45.5%)	68 (49.6%)		17 (47.2%)	10 (35.7%)	
Treatment			0.037			0.152
RFA	5 (7.6%)	21 (15.3%)		9 (25.0%)	11 (39.3%)	
TACE	26 (39.4%)	31 (22.6%)		17 (47.2%)	6 (21.4%)	
RFA+TACE	28 (42.4%)	62 (45.3%)		6 (16.7%)	6 (21.4%)	
Resection	7 (10.6%)	23 (16.8%)		4 (11.1%)	3 (10.7%)	
HBV-DNA (positive)	23 (42.6%)	36 (33.0%)	0.232	14 (38.9%)	10 (35.7%)	0.067
Antiviral drugs	28 (49.1%)	63 (52.9%)	0.635	18 (50%)	16 (57.1%)	0.570
AFP (positive)	40 (60.6%)	53 (38.7%)	0.003	21 (58.3%)	9 (32.1%)	0.037
MELD score^a^	5.9 ± 4.5	5.1 ± 4.2	0.197	5.5 ± 2.4	4.8 ± 2.3	0.276
WBC^a^ (×10^9^/L)	4.3 ± 2.0	4.1 ± 2.0	0.409	4.1 ± 1.9	4.5 ± 1.8	0.414
HGB^a^ (g/L)	130.0 ± 21.5	126.3 ± 25.7	0.197	124.9 ± 22.1	127.0 ± 17.0	0.681
ALT^b^ (U/L)	29.7 (21.6, 45.5)	27.9 (19.0, 41.3)	0.258	28.0 (22.1, 39.2)	24.3 (19.1, 56.5)	0.892
TBil^b^ (μmol/L)	17.1 (12.5, 25.4)	17.2 (12.5, 25.6)	0.647	17.9 (12.4, 26.5)	15.5 (11.5, 28.5)	0.361
GGT^b^ (U/L)	41.0 (25.5, 79.6)	34.1 (21.0, 57.4)	0.023	46.1 (26.9, 93.5)	27.0 (18.0, 40.0)	0.004
TP^b^ (g/L)	67.2 (63.7, 69.9)	69.5 (64.6, 74.7)	0.032	66.5 ± 6.9	69.2 ± 8.4	0.156
ALB^b^ (g/L)	36.8 (31.3, 40.9)	38.20 (32.0, 42.5)	0.167	39.0 (30.5, 41.8)	40.0 (33.7, 41.8)	0.477
CR^a^ (μmol/L)	70.3 ± 16.6	69.6 ± 16.0	0.762	80.2 ± 29.6	68.6 ± 16.8	0.068
PT^a^ (s)	13.8 ± 2.8	13.5 ± 2.3	0.398	13.2 ± 2.4	12.6 ± 1.5	0.234
CD8^+^ T lymphocytes^a^	248.1 ± 135.9	321.2 ± 209.8	0.040	332.2 ± 186.7	421.6 ± 349.6	0.327

^a^ These values are presented as mean ± standard deviation.

^b^ These values are presented as range followed by 95% confidential interval in parentheses; other values are presented as numbers of patients followed by percentages in parentheses.

CTP, Child-Turcotte-Pugh; RFA, radiofrequency ablation; TACE, transarterial chemoembolization; HBV, hepatitis B virus; AFP, α-fetoprotein; MELD, Model for End-Stage Liver Disease; WBC, white blood cells; NLR, neutrophil–lymphocyte ratio; PLT, platelets; ALT, alanine transaminase; TBIL, total bilirubin; GGT, γ-glutamyl transpeptidase; TP, total proteins; ALB, albumin; CR, creatinine; PT, prothrombin time.

In the training cohort, patients who experienced HCC recurrence within 1 year had a higher rate of heavy drinking (*P *=* *0.029), higher CTP grades (*P *=* *0.025), a higher rate of treatment with TACE (*P *=* *0.037), higher GGT levels (*P *=* *0.023), lower TP levels (*P *=* *0.032) and higher rates of AFP positivity (*P *=* *0.003) than patients who did not have disease recurrence.

In the validation cohort, patients with HCC recurrence within 1 year were more commonly male (*P *=* *0.042) and had a higher rate of heavy drinking (*P *=* *0.019), higher GGT levels (*P *=* *0.004) and higher rates of AFP positivity (*P *=* *0.037) than patients without disease recurrence.

For patients without a history of heavy drinking, HBV-DNA level was not associated with outcome in either the training cohort (*P *=* *0.903, chi-squared test) or the validation cohort (*P *=* *0.744, chi-squared test).

### Predictors of post-operative recurrence of HCC

**Figure 1.**
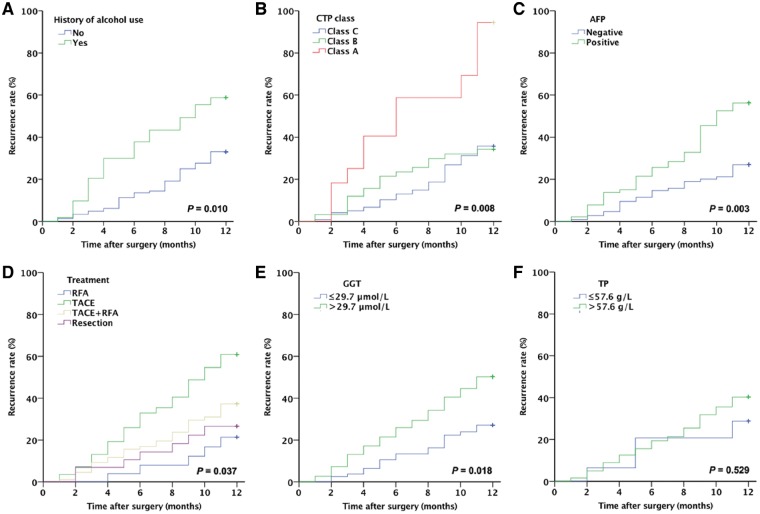
History of alcohol use, CTP class, AFP levels, GGT levels and treatment approach are associated with the 1-year recurrence rate of a single small HBV-related HCC. The curves were constructed using the Kaplan–Meier method and analyzed using the log-rank test for 1-year recurrence rate of single small HBV-related HCC (**A**) according to the history of heavy alcohol use; (**B**) according to CTP grade; (**C**) according to serum AFP positivity; (**D**) according to serum GGT levels; (**E**) according to serum TP level; and (**F**) according to the treatment approach. CTP, Child-Turcotte-Pugh grade; AFP, α-fetoprotein; GGT, γ-glutamyl transpeptidase; HBV, hepatitis B virus; HCC, hepatocellular carcinoma; TP, total protein.

The 1-year HCC recurrence rate was higher in patients with a history of alcoholism than in those without a history of alcoholism (44.4% vs. 28.2%, *P *=* *0.01; Figure 1A). The 1-year HCC recurrence rate was significantly higher in patients with CTP grade C than in those with CTP grade A (61.1% vs. 30.1%, *P *=* *0.008; Figure 1B). AFP-positive patients had a higher 1-year HCC recurrence rate than AFP-negative patients (43.0% vs. 23.6%, *P *=* *0.003; Figure 1C).

The cut-off values for GGT and TP were determined based on the Youden index. The 1-year HCC recurrence rate was significantly higher in patients with GGT >29.7 μmol/L than in those with GGT ≤29.7 μmol/L (*P *=* *0.018; Figure 1D). However, the 1-year HCC recurrence rate did not differ significantly between patients with TP >57.6 g/L and those with TP ≤57.6 g/L (*P *=* *0.529; Figure 1E).

The 1-year recurrence rate showed significant variation depending on the treatment used after the diagnosis of HCC (*P *=* *0.037). The lowest 1-year recurrence rate (19.2%) was observed in patients who underwent radiofrequency ablation (RFA), while the highest 1-year recurrence rate (45.6%) was observed in patients treated with TACE (*P *=* *0.019). The 1-year recurrence rate after liver resection (23.3%) was not significantly difference from that after RFA (*P *=* *0.654; Figure 1F).

These results indicate that certain demographic and clinical characteristics are associated with HCC recurrence within 1 year of surgery.

### Establishment of the scoring model

The results of the univariable Cox regression analyses showed that a history of alcoholism, serum levels of GGT, AFP and TP, and blood levels of CD8^+^ T lymphocytes were associated with 1-year HCC recurrence rate. The multivariable Cox regression analysis indicated that a history of alcoholism (HR, 1.813; 95% confidence interval [CI], 1.081–3.039; *P *=* *0.024), serum GGT level (HR, 1.006; 95% CI, 1.002–1.010; *P *=* *0.003), serum TP level (HR, 0.960; 95% CI, 0.929–0.992; *P *=* *0.014) and serum AFP level (HR, 2.073; 95% CI, 1.232–3.487; *P *=* *0.006) were independently associated with 1-year recurrence rate in patients with HBV-related HCC who had a single small primary tumor (Table 2).

**Table 2.** Cox regression analyses of the factors associated with the 1-year recurrence rate in patients with a single small HCC

	Univariable analysis			Multivariable analysis		
Variable	HR	*P*	95% CI	HR	*P*	95% CI
Sex	0.540	0.053	0.289–1.009			
Age	0.997	0.812	0.971–1.024			
Alcoholism history	1.889	0.013	1.143–3.120	1.813	0.024	1.081–3.039
WBC	1.049	0.424	0.934–1.178			
NLR	1.028	0.420	0.961–1.100			
PLT	0.997	0.299	0.992–1.002			
ALT	1.001	0.498	0.998–1.003			
GGT	1.008	0.000	1.004–1.012	1.006	0.003	1.002–1.010
TBIL	1.005	0.334	0.995–1.015			
TP	0.970	0.050	0.941–1.000	0.960	0.014	0.929–0.992
HBV-DNA	1.439	0.186	0.839–2.468			
PT	1.042	0.387	0.949–1.143			
AFP	1.809	0.019	1.250–3.359	2.073	0.006	1.232–3.487
CD8^+^ T lymphocytes	0.998	0.048	0.996–1.000			

HR, hazard ratio; 95% CI, 95% confidence interval; WBC, white blood cells; NLR, neutrophil–lymphocyte ratio; PLT, platelets; ALT, alanine transaminase; GGT, γ-glutamyl transpeptidase; TBIL, total bilirubin; TP, total proteins; HBV, hepatitis B virus; PT, prothrombin time; AFP, α-fetoprotein.

The Cox regression equation was: recurrence score (RS) = 0.595 × history of alcoholism (yes, 1; no, 0) + 0.006 × GGT − 0.041 × TP + 0.729 × AFP (positive, 1; negative, 0). The optimal cut-off value (specificity + sensitivity − 1) was calculated as −1.67. Therefore, RS = −1.67, RS < −1.67 and RS > −1.67 was taken to indicate average, low and high risk of HCC recurrence during the first year after surgery, respectively.

### Prognostic performance of the RS

**Figure 2.**
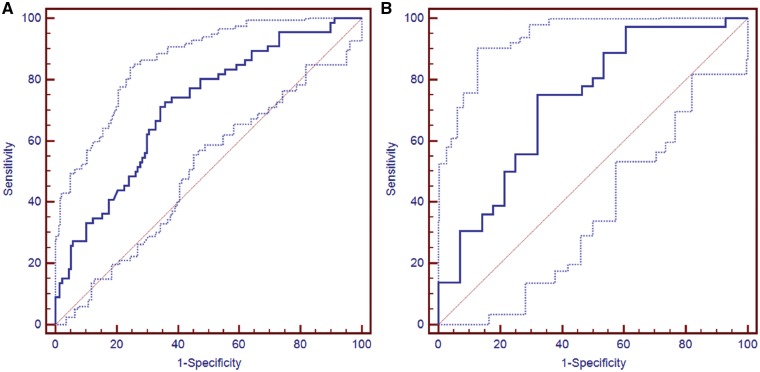
The recurrence score has a good prognostic performance in predicting the 1-year recurrence rate of a single small HBV-associated HCC. The patients in the training and validation cohorts were divided into high-risk and low-risk groups according to the cut-off value (−1.67) of the recurrence score. The prognostic performance was analyzed using ROC curve analysis; (**A**) the training cohort; (**B**) the validation cohort. HBV, hepatitis B virus; HCC, hepatocellular carcinoma; ROC, receiver operating characteristic; CI, confidence interval.

**Figure 3.**
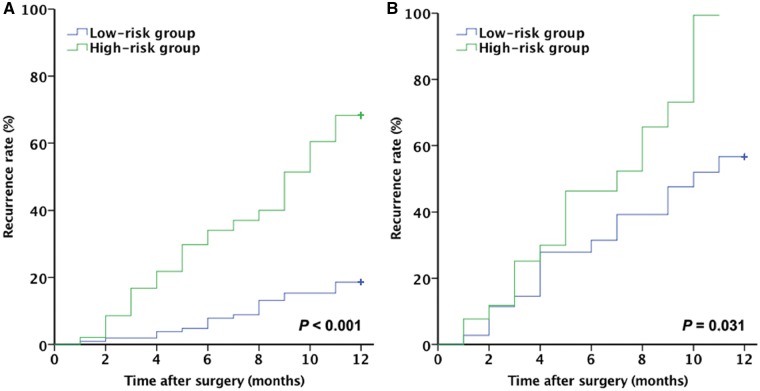
The survival rate differed between the low-risk and high-risk recurrence groups. The curves were constructed using the Kaplan–Meier method and analyzed using the log-rank test. One-year recurrence rates for the high-risk and low-risk groups are based on the recurrence score in (**A**) the training cohort and (**B**) the validation cohort

The AUC of the RS was 0.711 (95% CI, 0.643–0.772) in the training cohort and 0.727 (95% CI, 0.601–0.831) in the validation cohort (Figure 2). Patients in the training and validation cohorts were divided into high-risk and low-risk groups according to the optimal cut-off value for RS (−1.67). There were 106 patients with low HCC recurrence risk and 97 patients with high HCC recurrence risk in the training cohort, and the 1-year recurrence rate differed significantly between them (17.0% vs. 49.5%, *P *<* *0.001; Figure 3A). In the validation cohort, the 1-year recurrence rate was 43.2% in the low-risk group and 74.1% in the high-risk group (*P *=* *0.031; Figure 3B). These results indicate that a prognostic model can be built and used clinically to predict the prognosis of patients with HBV-related HCC who receive surgical treatment for a single small primary tumor.

## Discussion

The results showed that a history of alcoholism and serum levels of AFP, TP and GGT were independently associated with the 1-year recurrence rate of HBV-related HCC in patients treated for a single small primary tumor. The RS could provide a basis for clinicians to determine the post-operative HCC recurrence risk in these patients.

The factors influencing the post-operative recurrence of primary HBV-related HCC include tumor factors, peripheral vascular factors and liver function. Cheng *et al*. [23] reported that the independent risk factors for early recurrence after liver resection were tumor size >5 cm, tumor without a capsule and microvascular invasion, while the independent risk factors for late recurrence were cirrhosis and AFP >400 μg/L. Truant *et al*. [28] suggested that portal-vein invasion and tumor size >8 cm were independent risk factors for mortality in patients with HCC. Hirokawa *et al*. [29] concluded that HCC recurrence within 6 months after liver resection was associated with a lower survival rate. Moreover, in their study, vascular invasion and indocyanine green retention rate at 15 min (ICGR15) ≥16% were considered to be independent risk factors for early post-operative recurrence. Patients with HBV-related HCC who had a single small primary tumor were included in the present study; the results showed that AFP, GGT and a history of alcoholism were independent risk factors for HCC recurrence within 1 year after surgery, while TP was a protective factor. These results are consistent with those reported in previous studies [23, 28, 29]. These factors were used to build a scoring system that could allow closer surveillance of patients at higher risk of recurrence and earlier intervention to improve their prognosis.

Serum AFP level is an important parameter used in the detection of HCC and many studies have found that AFP is also a prognostic indicator in patients with HCC. Marubashi *et al*. [30] showed that the presence of AFP mRNA-expressing cells was an independent risk factor for post-operative recurrence in patients who received a liver transplant. Lu *et al*. [31] reported that AFP played an important role in promoting HCC metastasis. Kanda *et al*. [32] suggested that patients with HCC and an AFP level >100 μg/L were more prone to having post-operative recurrence and metastasis of HCC. Lee *et al*. [33] indicated that AFP and DCP levels predicted the survival of patients after TACE treatment. The present study confirmed that AFP levels were significantly increased in patients with HBV-associated HCC and a single small primary tumor who experienced recurrence during the first year after treatment than in those without recurrence. Furthermore, AFP was an independent risk factor for 1-year HCC recurrence in these patients.

Long-term heavy alcohol consumption can directly or indirectly damage the liver, causing hepatic fibrosis and liver cancer. Chavez *et al*. [34] confirmed that long-term chronic overdose of ethanol in mouse models could reduce the level of insulin-like growth factor-1 (IGF-1), affecting the proliferation of normal hepatocytes and promoting tumorigenesis. Purohit *et al*. [35] indicated that smoking and alcoholism promoted the occurrence of cancer. Takeshita *et al*. [36] showed that high doses of alcohol were associated with HCC. In the present study, a history of alcoholism was associated with post-operative recurrence in patients with HBV-associated HCC who had a single small primary tumor.

In normal individuals, GGT is mainly distributed in the cytoplasm of hepatocytes and intrahepatic bile duct epithelial cells. Thus, serum GGT is often increased in patients with non-alcoholic fatty liver disease and might be a surrogate marker of the association between metabolic factors and liver damage [37, 38]. A recent study performed in patients with HBV from Taiwan, China, showed that metabolic risk factors increased the risk of HCC development in the setting of chronic HBV hepatitis [39]. Investigations have shown that GGT levels are associated with the occurrence and prognosis of HCC [40–43]. The present study found that GGT levels were positively associated with the 1-year recurrence rate of single small HCCs, which is consistent with the results of the above studies.

TP mainly includes globulin and albumin. Deng *et al*. [44] showed that the albumin/globulin ratio was associated with the prognosis of HCC. Albumin and globulin are both secreted by the liver and the levels of these proteins in the blood represent the liver status and are associated with HCC prognosis [45, 46]. In the present study, TP level was an indicator of HCC recurrence in patients with small HCC, implying that TP is associated with the prognosis of HCC regardless of globulin or albumin reductions.

Many scoring systems are available for predicting the risk of HCC occurrence [15–19]. Nevertheless, HBV-related HCC has a specific natural history that differs from that of non-HBV-related HCC [3, 4]. The prognosis of HCC is also affected by whether early or late recurrence occurs after resection [20, 21]. The present study proposes an innovative scoring system that is specific to patients with HBV-related HCC who have a single small tumor. Additional studies are necessary to validate and improve this scoring system, for example by the inclusion of additional factors. Previous studies have reported that hyaluronic acid levels, the viral load of HBV and the liver inflammatory state are associated with HCC recurrence [47, 48], but these factors were not assessed in the present study.

This study also has some limitations. First, the study did not consider the tumor location, distance between the tumor and the portal vein, and the pathological features of the tumor. However, our research focused on serum markers that are easy to obtain in the clinical setting and the final model still had a high diagnostic value after validation. Second, the sample size of this study was small and the 95% CI of the AUC was wide. Although a large number of patients with HCC are seen at our institution, the present study aimed to establish a model specifically for patients with small HCCs, which limited the sample size. The sample size should be expanded in a future prospective cohort study. Third, the follow-up was only 1 year and the recurrence rates at 2 and 3 years were not available. Nevertheless, the recurrence rate was already high at 1 year (>30%), highlighting that early prevention and treatment are important for the survival of these patients. Fourth, the follow-up was carried out every 3–6 months or more frequently in cases of elevated AFP level or abnormal liver ultrasound, but the follow-up interval was not controlled due to the retrospective nature of the study; this may have introduced a follow-up bias. The recurrence rates for the two cohorts were significantly different. The hospital moved to a new site in 2008 and there were not as many patients in the first few years as in recent years. In addition, the new hospital has received more non-resident patients and patients with a more severe disease status. Finally, the model was based on patients from Beijing and its surroundings, so studies of other geographical areas are needed. In addition, no assessment of model calibration was performed because of the small sample size.

Radical treatments of HCC include resection and RFA. TACE is considered a palliative treatment and cannot achieve a curative outcome. Nevertheless, some patients in our study received TACE as the primary treatment for a variety of reasons, usually poor liver function and contraindications to surgery. This therapeutic approach is supported by the Chinese guidelines for the treatment of HCC [26]. The purpose of the present study was to explore the factors affecting post-operative 1-year HCC recurrence in patients with HBV-associated HCC who had a single small (≤3 cm) primary tumor and to suggest a scoring system for the prediction of recurrence. In the present study, the criteria for recurrence included the detection of new lesions suggestive of liver cancer in and around the original lesion or in other parts of the liver during follow-up. Based on this definition, the original lesion did not have to be removed.

## Conclusions

A history of alcoholism and serum levels of AFP, TP and GGT were independently associated with the 1-year recurrence rate of HBV-related HCC in patients with a single small (≤3 cm) primary tumor. Despite its limitations, the RS could provide an estimation of the post-operative 1-year HCC recurrence risk in these patients, but the score has to be validated in larger cohorts.

## Authors’ contributions

Z.Y.Y., X.B.W., and Y.Y.J. conceived of and designed the project. L.L.H., X.L.L., S.Z., and M.G.L collected the data. L.L.H. and X.L.L. analyzed the data and drafted the manuscript. All authors read and approved the final manuscript.

## Funding

This study was supported by the Beijing Municipal Science and Technology Commission [No. Z171100001017082], the Special Fund of Capital Health Research and Development [No. 2016–2-2171], the Fund for Beijing Science & Technology Development of TCM [No. JJ2016-14] and the Science and Technology Project of Beijing Municipal Education Commission [No. SQKM201610025026].
